# Idiopathic myointimal hyperplasia of mesenteric veins: radiological evaluation using CT angiography

**DOI:** 10.1093/bjrcr/uaad009

**Published:** 2023-12-13

**Authors:** Fumio Morimura, Hiromi Edo, Takafumi Niwa, Hiroaki Sugiura, Yohsuke Suyama, Soya Okazaki, Kazuyuki Narimatsu, Hiroki Ohno, Koichi Okamoto, Hideki Ueno, Shinya Yoshimatsu, Kosuke Miyai, Kohei Hamamoto, Hiroshi Shinmoto

**Affiliations:** Department of Radiology, National Defense Medical College, Saitama 3598513, Japan; Department of Radiology, National Defense Medical College, Saitama 3598513, Japan; Department of Radiology, National Defense Medical College, Saitama 3598513, Japan; Department of Radiology, National Defense Medical College, Saitama 3598513, Japan; Department of Radiology, National Defense Medical College, Saitama 3598513, Japan; Department of Internal Medicine, National Defense Medical College, Saitama 3598513, Japan; Department of Internal Medicine, National Defense Medical College, Saitama 3598513, Japan; Department of Surgery, National Defense Medical College, Saitama 3598513, Japan; Department of Surgery, National Defense Medical College, Saitama 3598513, Japan; Department of Surgery, National Defense Medical College, Saitama 3598513, Japan; Department of Basic Pathology, National Defense Medical College, Saitama 3598513, Japan; Department of Basic Pathology, National Defense Medical College, Saitama 3598513, Japan; Department of Radiology, Jichi Medical University, Tochigi 3290498, Japan; Department of Radiology, National Defense Medical College, Saitama 3598513, Japan

**Keywords:** idiopathic myointimal hyperplasia of mesenteric veins, CT angiography, bowel ischaemia

## Abstract

A 44-year-old man presented with a chief complaint of constipation. Initial contrast-enhanced CT showed extensive bowel wall thickening, mainly in the left colon, with a thin cord-like inferior mesenteric vein (IMV), in contrast to ectatic mesenteric venous branches, suggesting bowel ischaemia owing to venous stasis. One month later, at the time of symptom exacerbation, CT angiography showed a cord-like IMV and ectatic mesenteric venous branches with early enhancement, suggesting the presence of an arteriovenous fistula (AVF). Owing to the progression of bowel ischaemia and necrosis with peritonitis, emergency surgery was performed. Surgical specimens showed focal myointimal hyperplasia of the proximal mesenteric veins in both ischaemic and non-ischaemic lesions of the resected colon, thus leading to the diagnosis of idiopathic myointimal hyperplasia of mesenteric veins (IMHMV) when combined with the clinical and imaging findings. IMHMV is a bowel ischaemic disease caused by non-thrombotic venous obstruction that requires bowel resection and has been suggested to be associated with AVF. Cord-like IMV and AVF in the mesentery are important CT findings that characterize IMHMV. CT angiography is useful in diagnosing IMHMV.

## Clinical presentation

A 44-year-old man with a chief complaint of constipation for several weeks without abdominal pain and fever was referred to our hospital for further evaluation. On physical examination, a hard palpable mass was observed in the lower left to mid-abdomen with local warmth. White blood cell (WBC) count and C-reactive protein (CRP) were 6500/μL and 2.7 mg/dL, respectively. Colonoscopy performed at another hospital revealed significant oedema of the sigmoid colon mucosa and narrowing of the lumen, which made it difficult to pass the scope through the colon. The patient had similar symptoms 10 years prior and had undergone colonoscopy; however, no abnormal findings were noted. The past medical history included medications for hypertension. There was no other medical or medication history, including herbal medications, significant dietary history, or surgical history.

## Investigations/imaging findings

**Figure 1.**
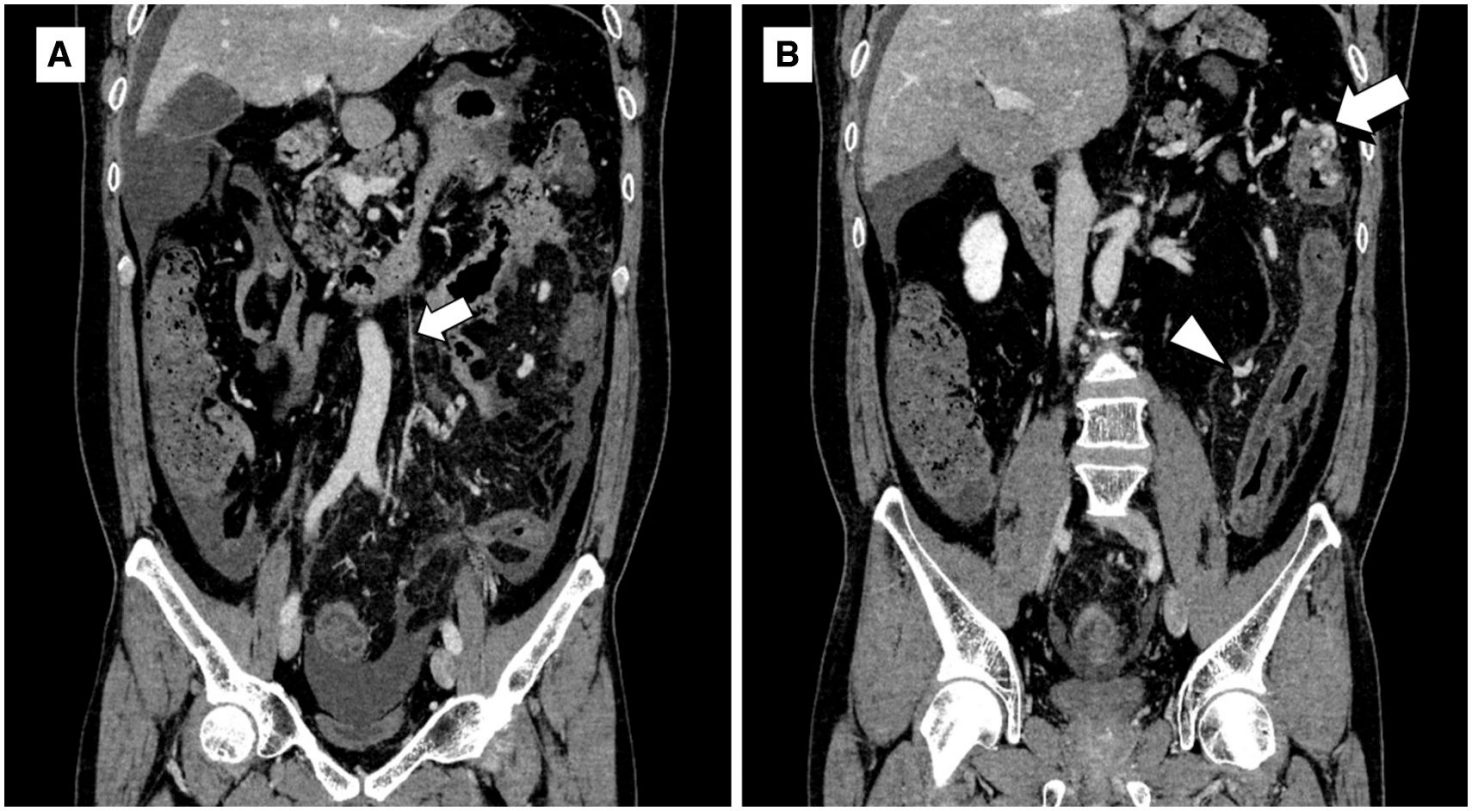
Coronal sections in the equilibrium phase on the initial contrast-enhanced computed tomography. (A) Thin cord-like IMV is depicted (arrow). (B) Ectatic mesenteric venous branches (arrowhead) and aneurysmal change of pericolic veins in the splenic flexure (arrow) are shown. Abbreviation: IMV = inferior mesenteric vein.

Initial contrast-enhanced CT showed an oedematous thickening of the bowel wall from the splenic flexure to the rectum. The CT also showed a thin cord-like inferior mesenteric vein (IMV) in contrast to the ectatic mesenteric venous branches, whereas the inferior mesenteric artery (IMA) was patent, suggesting that venous stasis owing to venous occlusion was associated with bowel ischaemia (Figure 1). There were no apparent thrombi in either the arteries or veins, arteriosclerotic changes, or calcifications along the veins and colonic walls.

Clinically, inflammatory bowel disease (IBD) and eosinophilic enteritis were suspected as differential diagnoses owing to the presence of ascites. However, the endoscopic biopsy specimen showed only a mildly inflamed mucosa without significant eosinophilic infiltration, which was incompatible with IBD or eosinophilic enteritis.

The patient was managed with careful observation because he had bowel movements, no abdominal pain, and showed only mild inflammation.

One month later, the patient complained of lower abdominal pain for 1 week and was taking non-steroidal anti-inflammatory drugs for self-medication. Physical examination revealed tenderness and guarding of the lower left abdomen. The body temperature was elevated to 38.0°C. WBC count and CRP were elevated to 18600/μL and 20.5 mg/dL, respectively.

**Figure 2.**
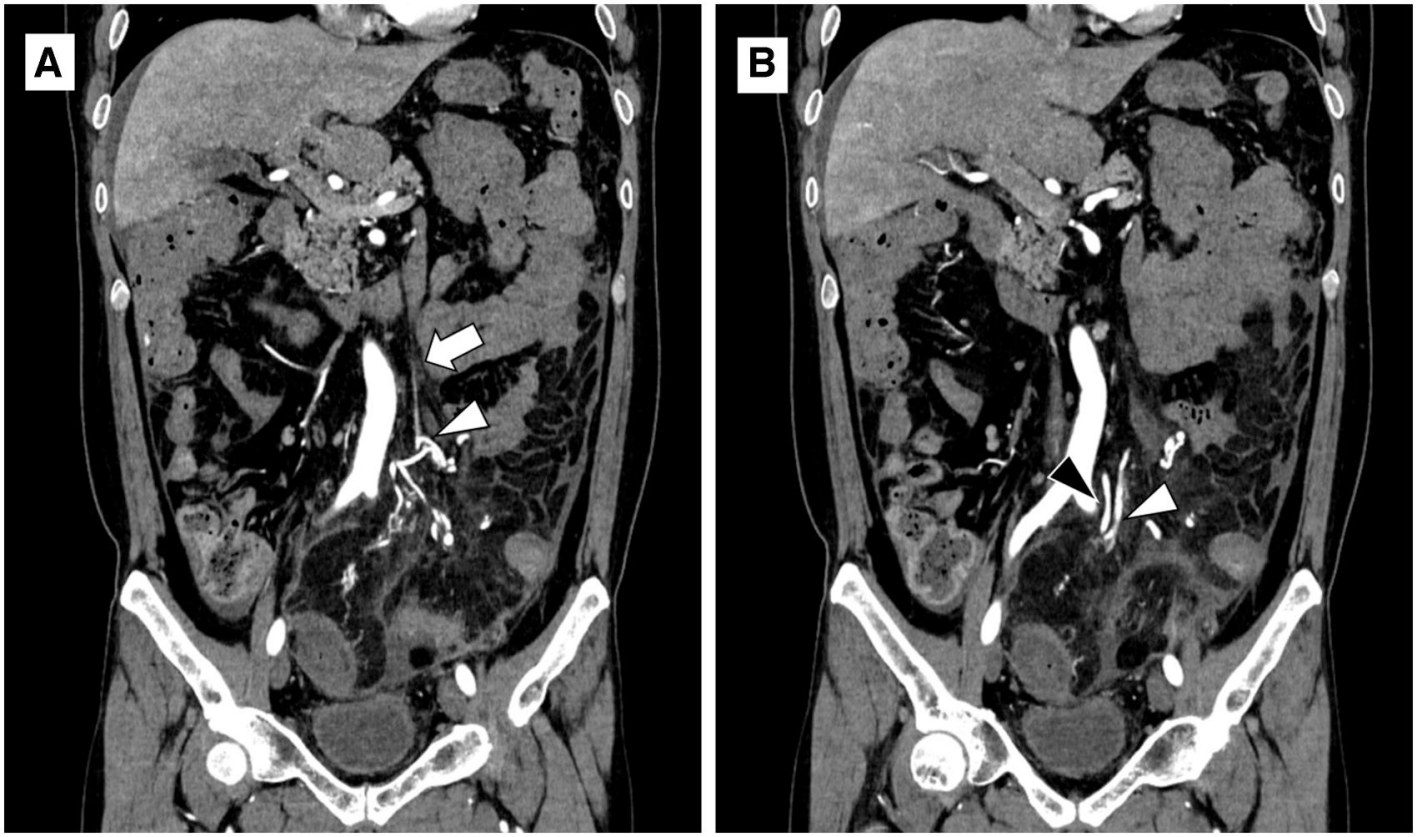
Coronal sections of CT angiography at the time of the patient’s symptom exacerbation (1 month after the initial contrast-enhanced CT). (A) Cord-like IMV is depicted (arrow), and the mesenteric venous branches (arrowhead) are enhanced in the arterial phase, which suggests an AVF in the mesentery. (B) The mesenteric venous branch (white arrowhead) runs along the IMA branch (black arrowhead) with comparable contrast enhancement in the arterial phase. The aneurysmal change of pericolic veins in the splenic flexure shown on the initial contrast-enhanced CT was not visualized in this CT angiography (not shown). Abbreviations: IMA = inferior mesenteric artery, IMV = inferior mesenteric vein, AVF = arteriovenous fistula, CT = computed tomography.

**Figure 3.**
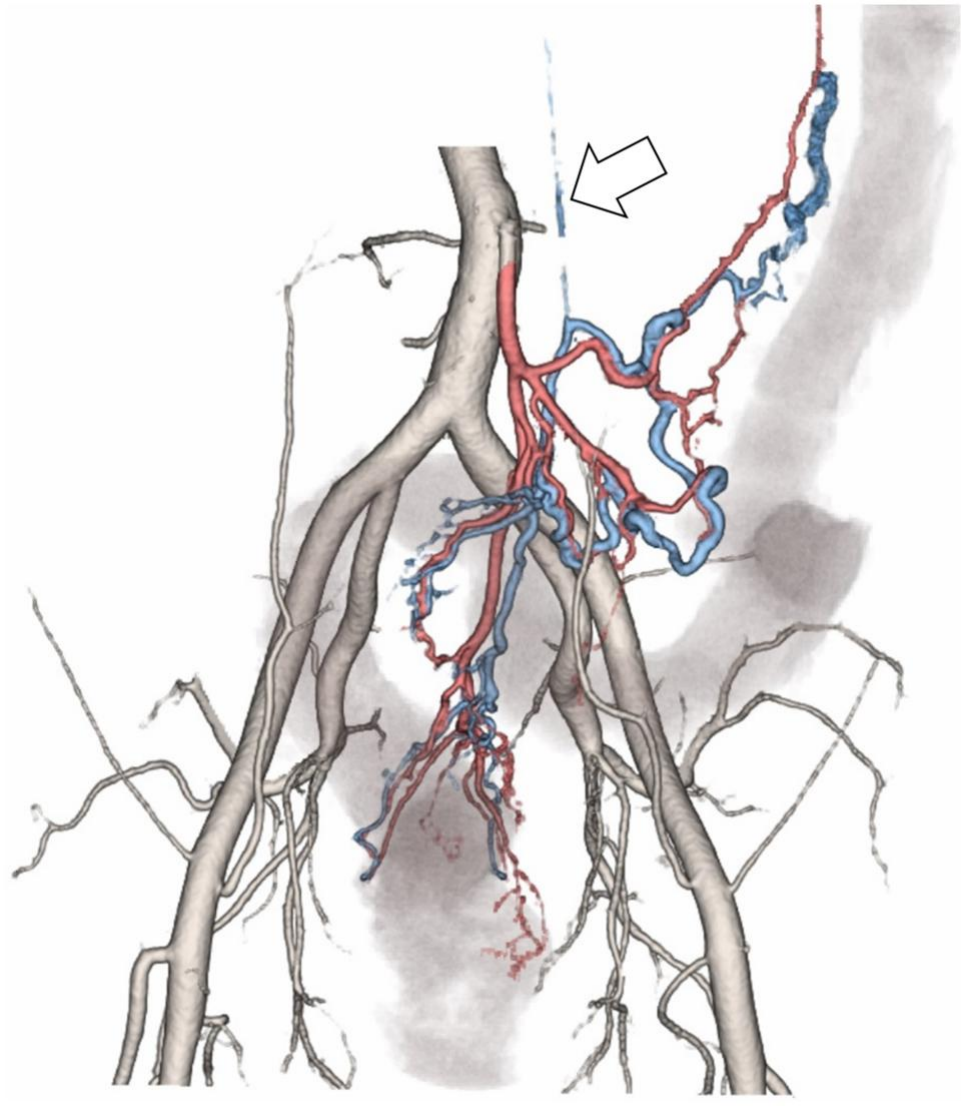
Volume rendering reformatted image on the CT angiography. The IMA and mesenteric arterial branches are shown in red. The IMV and the mesenteric venous branches are shown in blue. The descending and rectosigmoid colons are shown in grey. The arrow indicates the cord-like IMV. The ectatic mesenteric venous branches, which follow an anatomical course along the mesenteric arterial branches, are enhanced in the arterial phase, suggesting the presence of an AVF. Abbreviations: IMA = inferior mesenteric artery, IMV = inferior mesenteric vein, AVF = arteriovenous fistula, CT = computed tomography.

CT angiography was performed because vascular abnormalities, such as cord-like IMV and ectatic mesenteric venous branches, were suspected to be associated with the observed pathophysiology changes in the previous CT. Contrast-enhanced CT revealed oedematous bowel wall thickening extending into the mid-transverse colon. Poor mural enhancement is observed in the sigmoid colon, suggesting bowel necrosis. Peritoneal thickening was also observed. In the arterial phase of CT, marked enhancement was observed in the ectatic mesenteric venous branches, suggesting the presence of an arteriovenous fistula (AVF) (Figures 2 and 3).

## Differential diagnosis

The differential diagnosis of venous non-thrombotic bowel ischaemia in our case included the following:

- mesenteric inflammatory veno-occlusive disease (MIVOD)
- early stage of phlebosclerotic colitis (PC)
- idiopathic myointimal hyperplasia of mesenteric veins (IMHMV)

## Treatment

**Figure 4.**
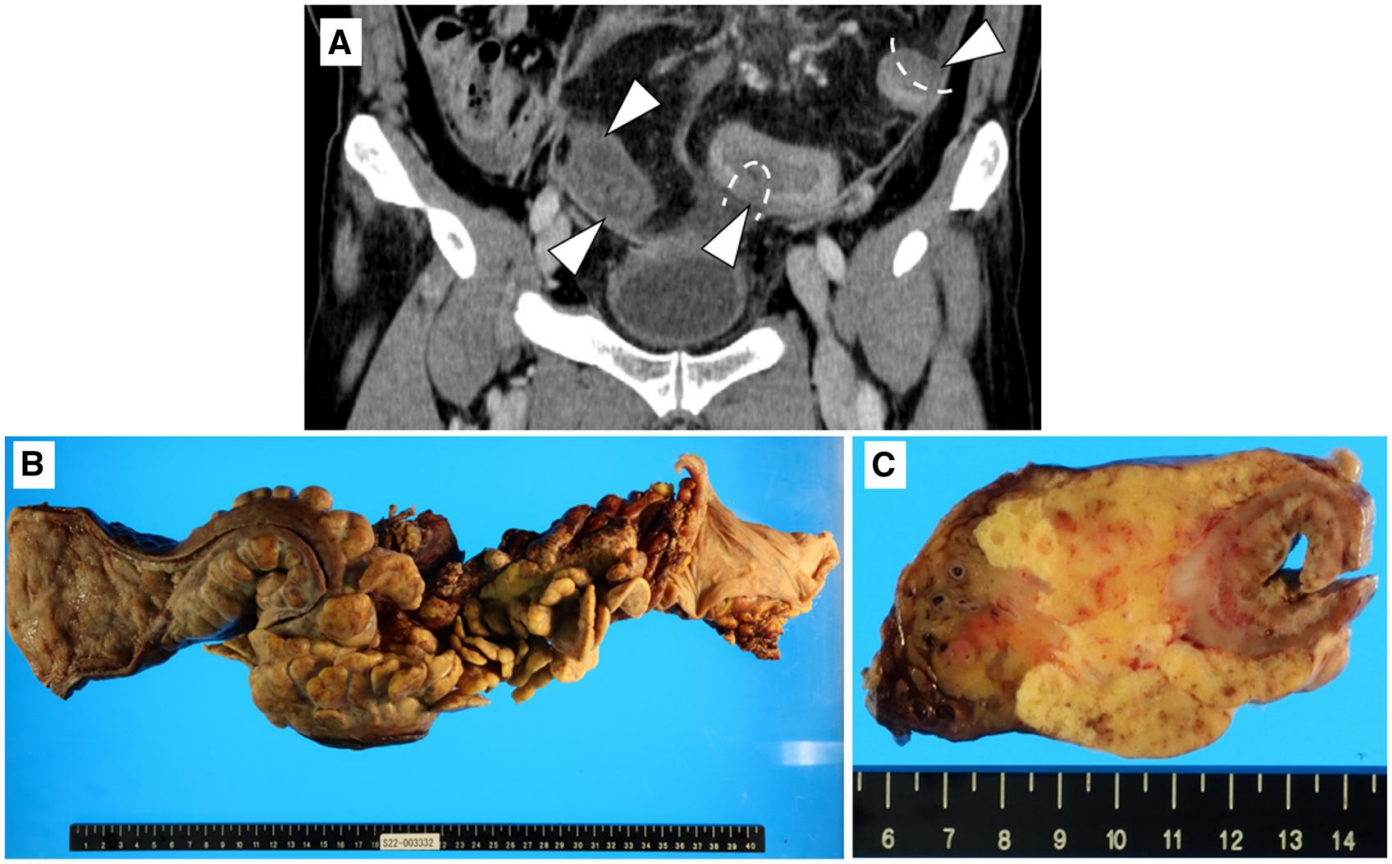
(A) A coronal section of the patient’s CT scan in the equilibrium phase at the time of symptom exacerbation. In the sigmoid colon, areas of poor contrast were observed in some parts of the bowel wall (arrowheads), suggesting bowel necrosis. (B) The gross image of the resected sigmoid colon. (C) The cross-sectional image of the resected specimen of the sigmoid colon. Total circumferential bowel necrosis is observed.

**Figure 5.**
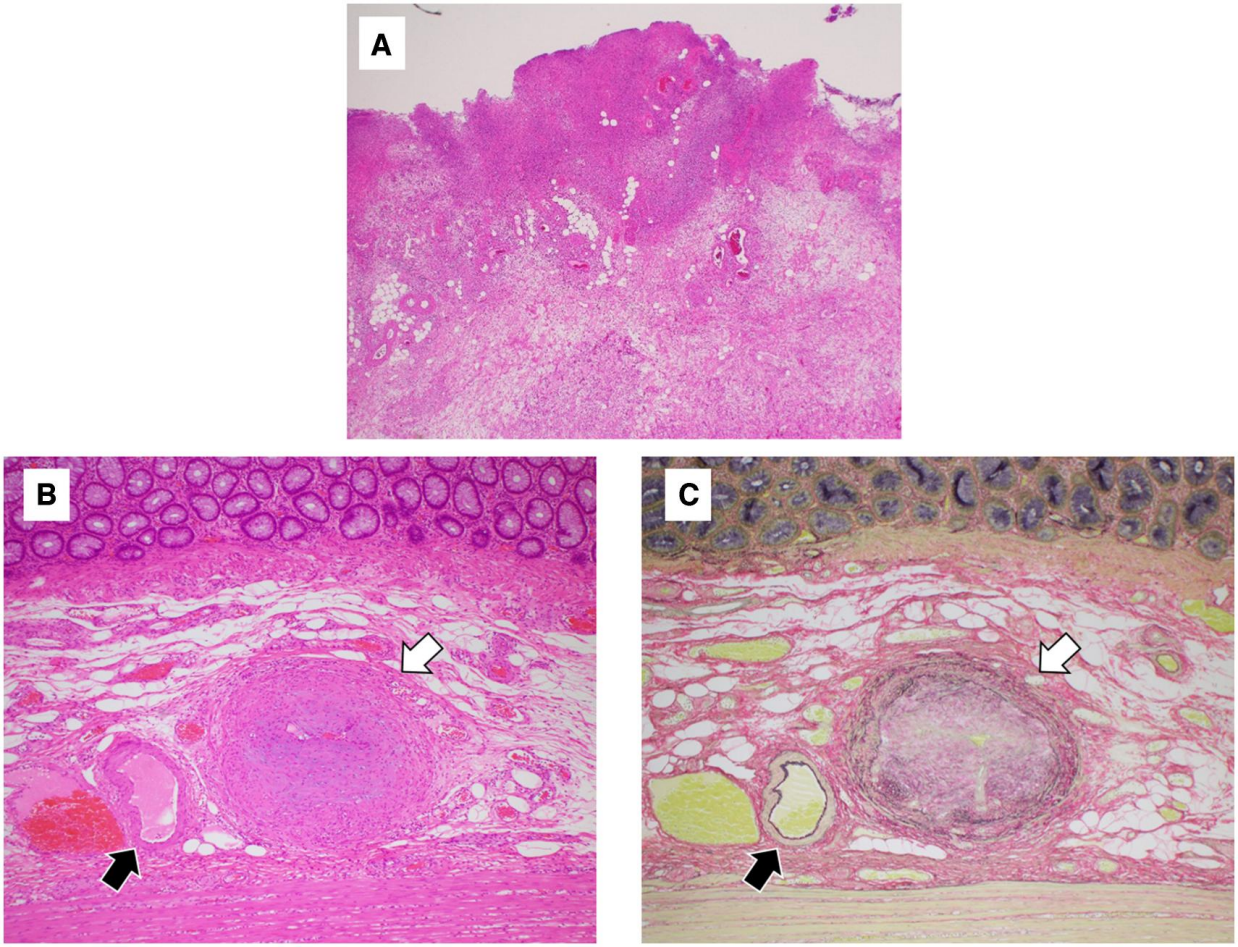
(A) Haematoxylin & eosin (H&E) staining shows broad ischaemic necrosis of the bowel mucosa. (B) H&E staining. A submucosal vein (white arrow) exhibits marked luminal narrowing due to myointimal hyperplasia in contrast with a nearby non-stenotic artery (black arrow). The covering mucosa is non-ischaemic. (C) Elastica van Gieson (EVG) staining. The internal elastic lamina of the artery (black arrow) is clearly stained, and that of the thickened vein (white arrow) is obscure due to myogenic proliferation in its intima.

Owing to the progression of bowel ischaemia and necrosis with peritonitis, emergency left hemicolectomy and proctectomy with transverse colostomy were performed. In the surgical specimen, almost the entire resected colon was oedematous, with focal mural necrosis (Figure 4). In both ischaemic and non-ischaemic lesions of the resected colon, at least focal muscular thickening of the intima was observed in the submucosal, subserosal, and mesenteric veins, which was considered to be the cause of bowel ischaemia (Figure 5). Although venous wall fibrosis was partly detected, phlebitis or venous wall calcification was not observed. Microscopically, chronic thrombi and recanalization were found only in the ischaemic lesions of the resected colon and were considered secondary changes.

## Outcome and follow-up

Based on the clinical manifestations and course, radiological findings, including cord-like IMV and AVF in the mesentery on CT angiography, and characteristic mesenteric vein pathology, IMHMV was the final multidisciplinary diagnosis. The patient was discharged on postoperative day 11 without complications, and there was no evidence of recurrence 1 year after surgery.

## Discussion

The 2 main causes of bowel ischaemia are arterial and venous. Arterial thrombotic disease is the most common cause of bowel ischaemia. Venous caused bowel ischaemia is less common and is typically thrombotic. Non-thrombotic causes are rare and include systemic vasculitis, such as systemic lupus erythematosus and Behçet’s disease, localized phlebitis called MIVOD, and non-inflammatory causes, such as PC and IMHMV. In particular, MIVOD and IMHMV require bowel resection. Therefore, it is important to distinguish MIVOD and IMHMV from other bowel ischaemias that can be treated conservatively.

IMHMV is an extremely rare disease, first described by Genta and Haggit in 1991,^1^ who reported striking intimal thickening involving the small mesenteric veins and their intramural branches. This report suggested that intimal thickening was caused by the proliferation of smooth muscle cells in a proteoglycan matrix and that elastic staining was required to differentiate thickened veins from arteries.^1^ In general, IMHMV has the following features: (1) young, previously healthy male patient; (2) segmental rectosigmoid colon involvement; (3) relatively protracted clinical course; (4) endoscopic and clinical impression of IBD; (5) endoscopic biopsy findings not compatible with IBD; (6) clinical complications necessitating segmental resection; (7) typical mesenteric vein pathology; and (8) uneventful follow-up with no recurrence.^2^ The present case fulfilled all of these criteria. The aetiology of IMHMV remains unknown. It has been hypothesized that intermittent torsion and stretching of the highly mobile sigmoid colon may have been contributed to it.^2^

**Figure 6.**
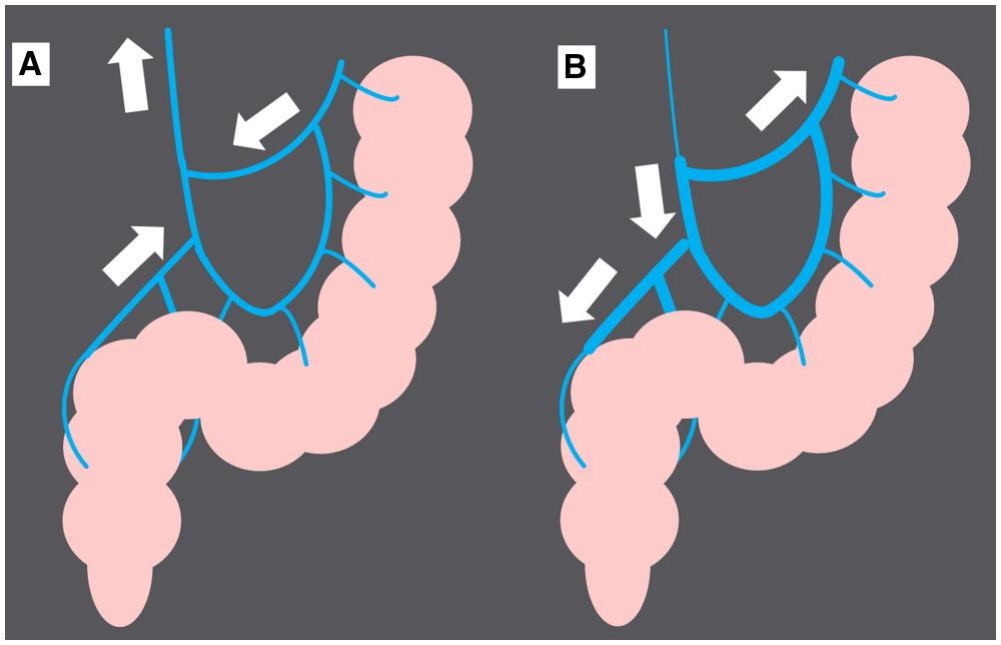
Schematic figure of IMV and rectosigmoid colon. The blue liner structure represents IMV, and the pink ductal structure represents the rectosigmoid colon. The white arrow represents venous blood flow. (A) Normal IMV. Normal hepatopetal blood flow is shown. (B) Abnormal IMV in IMHMV cases. The cord-like IMV and mesenteric venous branches are visualized. Abnormal hepatofugal flow owing to the cord-like IMV is shown. Abbreviations: IMV = inferior mesenteric vein, IMHMV = idiopathic myointimal hyperplasia of mesenteric veins.

Two characteristic imaging findings have been reported in IMHMV.^3–6^ First, a cord-like IMV has been reported as a feature of IMHMV.^3^ Although the affected area of the colon in IMHMV is compatible with ischaemic colitis, the cord-like IMV suggests that IMHMV is not just ischaemic colitis. In the present case, cord-like IMV was initially assumed to have developed after the thrombus disappearance; however, the hypercoagulable workup showed no abnormalities. Therefore, cord-like IMV is considered the cause of bowel ischaemia. The report referred to aneurysmal changes in the pericolic veins,^3^ which were also observed in the splenic flexure in this case on the initial CT. Aneurysmal changes in the pericolic veins may reflect venous stasis owing to the cord-like IMV and hepatofugal blood flow in the mesenteric venous branches (Figure 6). Secondly, an arteriovenous connection has been reported angiographically in IMHMV.^4^ The presence of AVF in the mesentery has been suggested since the first case report of IMHMV,^1^ but it has been difficult to prove it pathologically. A previous histological study has suggested that arterial pressure is required for the formation of myointimal hyperplasia.^7^ There are several other case reports that include imaging evaluations with angiography; however, in many of these reports, AVF remained undetected.^5^^,^^6^ Using angiographic evaluation, ectatic mesenteric vein branches may be recognized as arteries owing to hepatofugal blood flow in the early phase. It should also be noted that the IMV in patients with IMHMV may not be visible on angiography because of its thin, cord-like form, lack of early venous drainage, and lack of delayed contrast filling. In the present case, CT angiography was chosen instead of angiography to evaluate vascular abnormalities. Compared with angiography, CT angiography is more useful in the diagnosis of IMHMV because it is less invasive and can show the anatomic course of the IMA and IMV, abnormal enhancement of the mesenteric venous branches in the arterial phase, and cord-like IMV in the equilibrium phase without visible enhancement. Most previously reported IMHMV cases ultimately result in surgery,^8^ in contrast to ischaemic colitis, which is often treated conservatively. Patients with IMHMV should be evaluated at an early stage with CT angiography to minimize the need for surgical resection.

In the present case, MIVOD and PC were considered in the differential diagnosis of venous non-thrombotic bowel ischaemia because there were no other symptoms suggestive of systemic vasculitis. Due to no pathological evidence of phlebitis, MIVOD was excluded. In 2 previously reported MIVOD cases, a striking paucity of larger draining veins with a diminutive/irregular appearance of the IMV have been shown,^9^ which is similar to the cord-like IMV in IMHMV. AVF in the mesentery has also been reported in one MIVOD case.^10^ These 3 reported MIVOD cases demonstrated pathological myointimal hyperplasia. It has also been suggested that IMHMV is the presentation of a more advanced stage of MIVOD and that there is an overlap between the 2, although they are considered separate entities depending on whether vasculitis is present.^9^ Therefore, we believe that cord-like IMV and AVF in the mesentery are characteristic of IMHMV. PC is characterized by a predilection for the right side of the colon, calcification along the venous wall, and association with herbal medications (especially Sanshishi), which were not evident in this case. Although there is one report of PC, including a left-sided case with only slight calcification of the submucosal veins,^11^ no other PC cases have been reported to be left-sided, and the present case presented with no calcification both radiologically and pathologically. Therefore, IMHMV was considered to be the most appropriate diagnosis in this case.

## Conclusions

IMHMV is a bowel ischaemia due to non-thrombotic venous obstruction, the treatment of which mostly requires bowel resection. Radiologists should be aware of the characteristic findings of IMHMV. Cord-like IMV and AVF in the mesentery with bowel ischaemia are findings suggestive of IMHMV, and contrast-enhanced CT with CT angiography is necessary for detection.

## Learning points

- IMHMV causes non-thrombotic venous bowel ischaemia, which mostly requires bowel resection.
- IMHMV is characterized by striking intimal thickening involving the small mesenteric veins and their intramural branches, mostly affecting the left side of the colon. Elastic staining is required to differentiate between thickened veins and arteries.
- The characteristic imaging findings of IMHMV are cord-like IMV and AVF in the mesentery with bowel ischaemia. Because cord-like IMV cannot be depicted by angiography, CT angiography, which enables the simultaneous evaluation of cord-like IMV and AVF in the mesentery, is necessary for the diagnosis of IMHMV.
- If thickening of the bowel wall of the left-sided colon, such as in ischaemic colitis, is observed on CT, it is important to search for cord-like IMV and, if present, to add CT angiography for the evaluation of AVF in the mesentery.
